# Relationship between changes in the course of COVID-19 and ratio of neutrophils-to-lymphocytes and related parameters in patients with severe *vs*. common disease

**DOI:** 10.1017/S0950268821000674

**Published:** 2021-03-29

**Authors:** Kun Wang, Xin Wang, Jiangdong Du, Chunling Liu, Yanan Jiang, Heqiu Zhang, Haiming Jiang, Qiang Fu

**Affiliations:** 1 School of Pharmacy, Binzhou Medical University, Yantai, China; 2 Department of Clinical Laboratory, Yantai City Hospital for Infectious Diseases, Yantai, China; 3 Department of Clinical Laboratory, Yantai Yuhuangding Hospital, Yantai, China; 4 Intensive Care Unit, Yantai Affiliated Hospital of Binzhou Medical University, Yantai, China; 5 Shandong Cellogene Pharmaceutics Co. Ltd, Yantai, China

**Keywords:** Neutrophil-to-lymphocyte ratio, procalcitonin, SARS-CoV-2

## Abstract

To assess the relationship between the neutrophil-to-lymphocyte ratio (NLR) and related parameters to the severity of coronavirus disease 2019 (COVID-19) symptoms. Clinical data from 38 COVID-19 patients who were diagnosed, treated and discharged from the Qishan Hospital in Yantai over the period from January to February 2020 were analysed. NLR and procalcitonin (PCT) were determined in the first and fourth weeks after their admission, along with the clinical characteristics and laboratory test results of these patients. Based on results as obtained on the first and fourth weeks after admission, five indices consisting of NLR, white blood cells, neutrophils, lymphocytes (LY) and monocytes (MON) were selected to generate receiver operating characteristic curves, while optimal cutoff values, sensitivities and specificities were obtained according to the Yuden index. Statistically significant differences in neutrophils, LY and the NLR were present in the severe *vs.* moderate COVID-19 group from the first to the fourth week of their hospitalisation. The cut-off value of NLR for predicting the severity of COVID-19 was 4.425, with a sensitivity of 0.855 and a specificity of 0.979. A statistically significant positive correlation was present between PCT and NLR in the severe group as determined within the first week of admission. NLR can serve as a predictor of COVID-19 disease severity as patients' progress from the first to the fourth week of their hospitalisation. The statistically significant positive correlation between levels of NLR and PCT in severe patients indicated that increases in NLR were accompanied with gradual increases in PCT.

## Introduction

Coronavirus disease 2019 (COVID-19) is an acute respiratory infectious disease resulting from a severe, acute respiratory syndrome coronavirus 2 (SARS-CoV-2) infection [1]. The main route of transmission of this highly infectious virus involves respiratory droplets and close contact. The clinical scope of SARS-CoV-2 infection seems to be very broad, and can include asymptomatic infection, mild upper respiratory disease, severe viral pneumonia with respiratory failure and even death [2]. The most mild/moderate disease initial symptoms of COVID-19 consist of fatigue, dry cough and high fever, while diarrhoea, haemoptysis and headache are rare, and only a very small number of patients show loss of taste or smell [3]. In the later stages of infection, patients with COVID-19 may develop secondary bacterial infections [4]. There is evidence that peripheral blood neutrophils of patients with severe bacterial infection, as found with COVID-19, are affected by cytokines. Under such conditions, apoptosis of neutrophils is markedly inhibited, leading to a significant increase in the number of peripheral blood neutrophils [5].

Procalcitonin (PCT), C-reactive protein (CRP) and the neutrophil-to-lymphocyte ratio (NLR) have all been shown to serve as important markers of inflammation [6]. These markers, in particular, PCT and CRP, have been studied in patients with neutropenia. Inflammatory factors are also critical in the development of COVID-19. For example, Li *et al*. [7] reported that serum CRP levels in patients with severe COVID-19 were higher than those of patients with moderate COVID-19. Peng *et al*. [8] found that the serum PCT and CRP levels of patients with severe COVID-19-related cardiovascular disease were higher than those of mild/moderate disease patients. NLR is a simple biomarker of inflammation that can be measured in routine haematology [9]. The NLR may also provide an important index of this condition, as increases in NLR are associated with clinical deterioration and mortality in COVID-19 patients [10]. Liu *et al*. [11] found that NLR could serve as an early-stage predictor of COVID-19 in patients who were likely to develop a critical manifestation of this disease, with patients aged ⩾50 and an NLR ⩾3.13 being at greatest risk.

Currently, there are a number of studies relating to NLR, PCT and other related inflammatory markers to the mortality and prediction of initial degree of COVID-19 affliction. However, whether NLR can serve as a predictor of disease severity during its development and in the changes that can occur in this disease remain to be studied. Moreover, the relationship between NLR and PCT as predictors of COVID-19 needs to be assessed. In this study, we examined the relationship between NLR and the severity of COVID-19 and compared these with that of PCT in these patients as assessed from their first to fourth week of hospitalisation.

## Methods

### Materials and methods

#### General information and research methods

A retrospective analysis was conducted on the clinical data of 38 COVID 19 patients. These patients were diagnosed, treated and discharged from the Qishan Hospital in Yantai City over the period from January to February 2020. As based on the ‘New Coronavirus Pneumonia Diagnosis and Treatment Plan (Trial Eigth Edition)’ of the National Health Commission, the clinical classification at admission consisted of one of three types. (1) Light type – mild clinical symptoms with no pneumonia on imaging. (2) mild/moderate disease type – fever with respiratory symptoms and presence of pneumonia on imaging. (3) Severe type − adults with any of the following: (1) respiratory distress, RR ⩾30 beats/min, (2) oxygen saturation of ⩽93% during inhalation at rest, (3) an arterial partial pressure of oxygen (PaO_2_)/fraction of inspired oxygen (FiO_2_) of ⩽300 mm Hg and (4) chest imaging indicating an obvious ⩾50% progression of infiltrations within 24–48 h. The 38 patients were allocated into either a severe or mild/moderate disease type according to the diagnosis described above and treatment plan.

#### Statistical analysis

All data are presented as means ± SDs. Independent sample *t* tests were used to compare the differences between the two groups. The statistical software SPSS version 24.0 for Windows (IBM, Armonk, NY, USA) was used for data analysis. Counting data were expressed as rate (%) and tested with use of Fisher's exact probability method. The correlation between two variables is represented by *r* value. A *P* < 0.05 was required for differences in results to be considered as statistically significant.

## Results

### General data analysis

Patients within the severe type (*n* = 5) were significantly (*P* < 0.05) older (60 ± 6 years) than that of the mild/moderate disease type (*n* = 33; 45 ± 2 years). One case (2.6%) within the severe type was admitted to the intensive care unit due to organ dysfunction and eventually died. Among these 38 COVID-19 patients, 11 (28.9%) had one or more additional conditions, such as hypertension (21.1%), diabetes (5.3%), heart disease (5.3%) and chronic lung disease (5.3%). All 33 cases of the ordinary type recovered and were discharged, with one case (2.6%) remaining affected with a sequelae of hyposmia upon discharge (Table 1).

**Table 1.** Basic clinical characteristics of patients with severe and mild/moderate disease COVID-19

Clinical features	Total people *n* = 38	Type		*P*
		Severe *n* = 5	Mild/moderate disease *n* = 33	
Age	47 ± 13	60 ± 6	45 ± 2	0.0199
Sex (%^a^)				0.651^b^
Male	22(57.9)	3(7.9)	19(50.0)	
Female	16(42.1)	2(5.3)	14(36.8)	
Underlying illnesses (%^a^)				
History of heart disease	2(5.3)	1(2.6)	1(2.6)	0.249
Hypertension	8(21.1)	2(5.3)	6(15.8)	0.279
Hyperlipidaemia	3(7.9)	0(0)	3(7.9)	1.000
Diabetes	2(5.3)	1(2.6)	1(2.6)	0.249
History of asthma	2(5.3)	1(2.6)	1(2.6)	0.249
History of hepatitis	2(5.3)	0(0)	2(5.3)	1.000
Prognosis (%)				
Death		1(2.6)	0(0)	NA^c^
Discharged		4(10.5)	33(86.8)	0.243
Sequelae		0(0)	1(2.6)	NA^c^

a All percentages shown here were compared to the total number of people.

b *P* value indicated the difference between the two groups of men and women.

c Due to the small number of people, comparisons between the two groups were not applicable.

### Laboratory analysis

One week after admission, compared with the mild/moderate disease type, the white blood cell (WBC) count and neutrophil (NEU) indicators of the severe type were significantly increased, while lymphocytes (LY) were significantly decreased and even showed lymph cytopenia. Monocyte (MON) counts are in the normal range in both groups, but the severe type (0.34 ± 0.05) is slightly lower than the mild/moderate disease type (0.48 ± 0.03), *P* < 0.05 is statistically significant. The NLR value of the severe type group is as high as (21.39 ± 4.61), while the NLR value of the mild/moderate disease type group is (2.14 ± 0.15), *P* < 0.0001 (Table 2).

**Table 2.** Comparison of laboratory results between severe and mild/moderate disease COVID-19 patients within the first week of admission

Blood parameters	Type		*t*^a^	*P*
	Severe	Mild/moderate disease		
White blood cell (×10^9^/l)	9.95 ± 4.84	5.21 ± 1.24	6.338	<0.001
Neutrophils (×10^9^/l)	8.98 ± 5.00	3.07 ± 1.05	7.868	<0.001
LY (×10^9^/l)	0.39 ± 0.22	1.60 ± 0.53	−9.586	<0.001
Monocyte (×10^9^/l)	0.34 ± 0.24	0.48 ± 0.18	−2.640	0.010
NLR^b^	21.39 ± 20.08	2.14 ± 1.01	6.626	<0.001

a The value of the *t* test between two groups.

b NLR: neutrophil-to-lymphocyte ratio.

After 4-weeks of treatment, there were no longer significant differences (*P* = 0.0532) in WBC between the two groups, with levels in both returning to normal values. NEU levels within the mild/moderate disease type (2.86 ± 0.94) remaining significantly increased (*P* = 0.0009) over that of the severe type (8.98 ± 1.15). While the number of LY (0.88 ± 0.11) with the severe type increased slightly from the fourth to the first week after admission, they remained significantly lower (*P* < 0.0001) than that of the mild/moderate disease type (1.58 ± 0.35). NLR values in both groups decreased significantly from the first to the fourth week, with this decrease being more obvious in the severe *vs.* mild/moderate disease type (*P* = 0.0002) (Table 3).

**Table 3.** Comparison of laboratory results between severe and mild/moderate disease COVID-19 patients within the fourth week of admission

Blood parameters	Type		*t*^a^	*P*
	Severe	Mild/moderate disease		
White blood cell (×10^9^/l)	6.13 ± 1.65	5.04 ± 1.24	2.022	0.053
Neutrophils (×10^9^/l)	4.82 ± 1.77	2.86 ± 0.94	3.747	0.001
LY (×10^9^/l)	0.88 ± 0.42	1.58 ± 0.35	−4.904	<0.001
Monocyte (×10^9^/l)	0.33 ± 0.25	0.45 ± 0.10	−1.706	0.100
NLR	7.32 ± 4.85	1.84 ± 0.55	4.352	<0.001

a The value of the *t* test between two groups.

### Relationship between the NLR and severity of the COVID-19 disease over the first-to-fourth weeks of admission

**Fig. 1.**
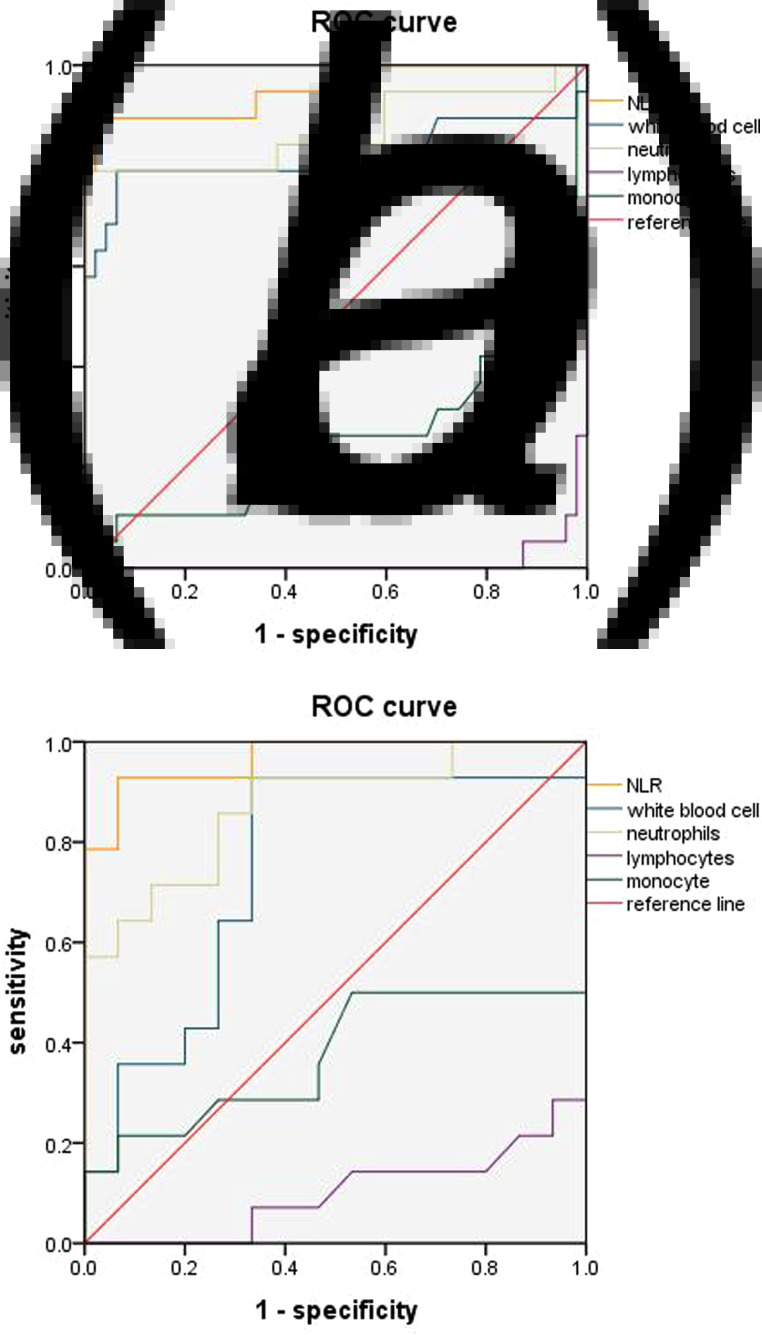
ROC curve analyses of NLR, WBC, NEU and LY. ROC curves of the four indicators in week 1 (a) and week 4 (b). The AUC at week 1 for NLR, WBC, NEU, LY and MON were 0.953, 0.815, 0.868, 0.012 and 0.267, respectively; and at week 4 were 0.967, 0.748, 0.871, 0.100 and 0.374, respectively. In the two courses, the AUC of NLR is the largest.

Five indices, consisting of NLR, WBC, NEU, LY and MON were used to assess differences between these two groups of COVID-19 patients as evaluated on weeks 1 and 4 after admission. To perform this analysis, we calculated the area under the curve (AUC) of the receiver operating characteristic (ROC) curve, screened the best index and obtained the best cut-off value, sensitivity and specificity according to the Yuden Index. The AUC at week 1 for NLR, WBC, NEU, LY and MON were 0.953, 0.815, 0.868, 0.012 and 0.267, respectively; and at week 4 were 0.967, 0.748, 0.871, 0.100 and 0.374, respectively. In the two courses, the AUC of NLR is the largest (Fig. 1a and b).

As determined at one week after admission, the best cut-off value of NLR for predicting the severity of COVID-19 was 4.425, with a sensitivity of 0.855 and specificity of 0.979. These results with NRL were greater than all other indices. At 4 weeks, NLR, WBC and NEU all showed similar sensitivities for predicting disease severity (0.929), but NLR showed the highest specificity (0.933) (Table 4).

**Table 4.** The best cut-off values, sensitivities and specificities of NLR, WBC and NEU

Index	The best cut-off value		Sensitivity		Specificity	
	1w	4w	1w	4w	1w	4w
NLR	4.425	2.510	0.855	0.929	0.979	0.933
White blood cell	6.840	5.010	0.789	0.929	0.936	0.667
Neutrophils	5.215	1.215	0.789	0.929	1	0.667

## Discussion

In December 2019, an outbreak of COVID-19 was reported, which spread globally and became a major emergency of international concern. The incubation period for human-to-human transmission was described as being 2–14 days, enabling for an expedient dissemination and receipt of droplets from contaminated hands or surfaces [12]. Most patients infected with SARS-CoV-2 experience a mild type of this illness and present with mild/moderate disease symptoms such as fever, cough and fatigue. A small number of infected patients progress to more severe cases involving an acute respiratory distress syndrome, with some of these developing a rapid onset multiple organ failure resulting in death, especially in elderly patients with comorbidities [13]. It now seems fairly well established that the hyperinflammatory response induced by SARS-CoV-2 is a major cause of disease severity and death in infected patients [14].

In this study we summarised the clinical data of all nucleic acid-positive hospitalised COVID-19 patients as seen at our hospital over the period from January to February 2020. One salient observation was that the severity of the patient's condition was related to age. The older the patient, the more likely they were to develop the severe type of this disease (*P* < 0.05). As various bodily functions are compromised in the elderly, the virus is more likely to invade structures such as the lungs and other organs, thus increasing the severity of this disease. While no statistically significant differences were found between the severity of the disease and any underlying diseases in the patients of our study, it should be noted that this may be attributable to the small sample size (*n* = 5) in our severe COVID-19 group. As COVID-19 progresses, the number of circulating neutrophils gradually increases and neutrophil extracellular traps (NETs), the extracellular network of neutrophils that release DNA/histone proteins to control infection, exacerbate the inflammation [15]. Neutrophils and LY represent key cellular components of the human host defense system directed against infection [16]. Some early observations − and knowledge of the previous epidemic of SARS-COV-1 − show that monocytes and pulmonary monocyte-derived macrophages promote cytokine storms, acute respiratory distress syndrome and disseminated peripheral tissue damage plays an early and key role in the progression of severe new coronary pneumonia [17]. Analysis of the laboratory results of our patients revealed that WBC, NEU, LY and MON were all significantly increased in the severe *vs.* general group as determined at one week after admission (*P* < 0.05). With the progression and treatment of this disease the significant differences in WBC and MON between the two groups were no longer present at 4 weeks after admission (*P* > 0.05). It has been reported that a mild/moderate disease feature of many COVID-19 patients is a decrease in circulating LY, which is especially prominent in severe patients [14]. This effect was similar to that obtained in our current study.

After reviewing the data on clinical characteristics and laboratory results it became clear that some notable differences between the first and fourth weeks of admission were present in the severe *vs.* mild/moderate disease COVID-19 patients. Accordingly, we examined the changes in levels of NLR, WBC, NEU, LY and MON over the course of this disease as determined at weeks 1 and 4 of their hospitalisation. The relationship regarding disease severity was analysed using ROC curves. The AUCs obtained for LY and MON were <0.50, and therefore could not be used as potential diagnostic biomarkers for subsequent analysis. However, we did find that levels of NLR, WBC and NEU were predictive of disease development at both time periods. Among these three, NLR showed the best sensitivity and specificity. The predictive effect of NLR on disease severity was more apparent in the first *vs.* fourth week. As based on the NLR cutoff value, patients with an NLR ⩾4.425 were more likely to develop a severe type of this disease. Therefore, in patients with a NLR value of >4.425 as determined at 1 week after admission, clinicians should be alerted to the potential for development of a severe type of this disease and initiate early interventions to prevent this eventuality.

**Fig. 2.**
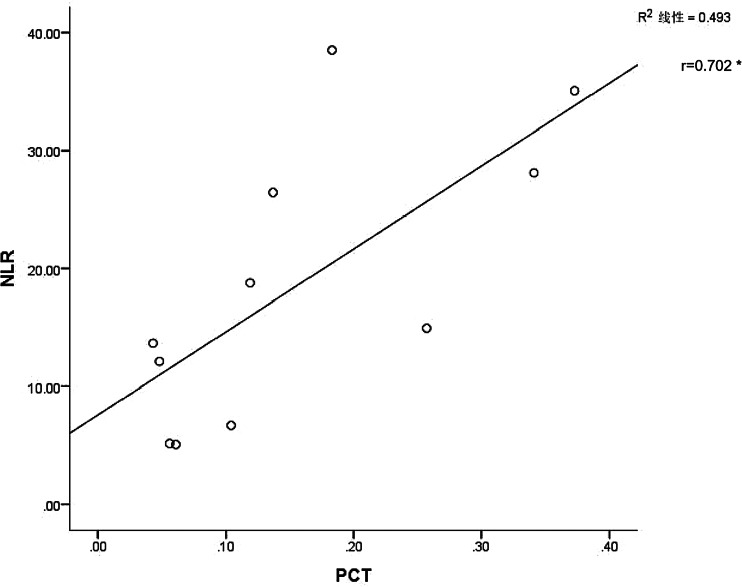
Linear analysis of NLR and PCT. Abscissa is the PCT value and ordinate the NLR value (*P < 0.05). The linear analysis of PCT and NLR of patients in the severe type within 1 week after admission showed that the NLR value would gradually increase with the increase of PCT, and there was a correlation between the two, the correlation coefficient *R* value was 0.702, which was statistically significant (*P* < 0.05).

As demonstrated using in vitro cell models, there is a delayed release of cytokines and chemokines in respiratory epithelial cells, dendritic cells (DCs) and macrophages during the early stages of SARS-CoV infection. These cells secrete low levels of antiviral factors interferons (IFNs) and high levels of proinflammatory cytokines (interleukin (IL)-1*β*, IL-6 and tumour necrosis factor (TNF)) and chemokines [18]. Another protein that can serve as an inflammatory marker is PCT, a peptide precursor of calcitonin that consists of 116 amino acids. Calcitonin is initially biosynthesised as PCT, which, under normal conditions, is found in low levels in the circulation [19]. PCT viral infection may be released under the action of proinflammatory mediators such as IL-6, TNF release or the like, which leads to elevated levels of peripheral blood [20]. As both NLR and PCT may be used as inflammatory markers during the development of COVID-19, we next examined whether any relationship may exist between the two. It was only possible to perform a linear analysis of PCT and NLR within the first week of admission for severe patients, as PCT values of mild/moderate disease patients throughout the course of the disease were <0.04, which is below the detection level of our assay. We found that increases in PCT were accompanied with gradual increases in NLR values, with these responses showing a statistically significant positive correlation (*r* = 0.702, *P* < 0.05) between these two variables (Fig. 2).

In summary, our results show that as the course of COVID-19 progresses, laboratory test results of severe patients gradually return to normal, especially with regard to changes in white blood cells and monocytes. NLR plays an important role throughout the course of the disease and can predict which patients will become critically ill as based upon determinations performed in the early stages of the disease [11]. It can also predict the development of the disease from the first to the fourth week of their hospitalisation; it also plays a significant role in predicting the prognosis in the later stage of the disease development. PCT is also an important inflammatory marker. Moreover, NLR can also serve as a predictor of disease development from the first to the fourth week of their hospitalisation and play a significant role in predicting disease prognosis in the later stages of disease development. While PCT is also an important inflammatory marker, limitations in the detectability of our assay precluded any in-depth analysis of this protein as related to these COVID-19 patients. Future investigation utilising animal models may be required to assess the role of PCT in COVID-19, which may also play an important role in the development of this disease.

## Data Availability

The data for the study is available by contacting the corresponding author upon request.
